# Disease Attribution to Multiple Exposures Using Aggregate Data

**DOI:** 10.2188/jea.JE20210084

**Published:** 2023-08-05

**Authors:** Wen-Chung Lee, Yun-Chun Wu

**Affiliations:** 1 Institute of Epidemiology and Preventive Medicine, College of Public Health, National Taiwan University, Taipei, Taiwan; 2 Innovation and Policy Center for Population Health and Sustainable Environment, College of Public Health, National Taiwan University, Taipei, Taiwan

**Keywords:** disease attribution, attributable fraction, burden of disease, interaction, causal pie model

## Abstract

**Background:**

Identifying which exposures cause disease and quantifying their impacts is essential in promoting and monitoring public health. When multiple exposures are involved, measuring individual contributions becomes challenging.

**Methods:**

The authors propose a disease attribution method based on aggregate data or summary statistics of individual-level data, possibly from multiple data sources.

**Results:**

Using the proposed method, the burden of disease is apportioned to the independent and interaction effects of each of its major risk factors and all the other factors as a whole. This scheme guarantees that 100% is the total share of the burden.

**Conclusion:**

The calculation is simple and straightforward; therefore, it is recommended for use in studies on disease burden.

## INTRODUCTION

Identifying which exposures cause disease and quantifying their impacts is essential in promoting and monitoring public health.^1^ For instance, when planning intervention strategies, health authorities may seek to compare the effectiveness of various intervention programs directed at removing a specific exposure or a combination of exposures in the population. Disease attribution also constituted an integral part of the Global Burden of Diseases, Injuries, and Risk Factors (GBD) Study,^2^^,^^3^ in which differences in burdens of and risk factors for diseases in various countries or regions were compared. A commonly used index for disease attribution is the population attributable fraction (PAF).^4^^–^^6^

When multiple exposures are involved, measuring individual contributions becomes challenging. For example, a summation of the PAFs for all exposures may be >100%. Studies have prevented this problem by using the causal pie model,^7^^–^^9^ among other methods.^10^^–^^17^ However, all these methods require individual-level data.

In this paper, we propose a method based on the causal pie model^1^^,^^7^^–^^9^^,^^18^ to attribute diseases to multiple exposures using aggregate data or summary statistics of individual-level data, possibly from multiple data sources. The goal is to produce the “causal pie weights” (CPWs), which quantify the contributions from different classes of causal pies (summing up exactly to 100%). Two examples will be given to demonstrate the methodology.

## METHODS

### Notations, assumptions, and derivations

We use two binary exposures, *X* and *Z*, to present the methodology. Let *p*_00_(*t*), *p*_10_(*t*), *p*_01_(*t*), and *p*_11_(*t*) denote the proportions at time *t* of people exposed to neither *X* nor *Z*, *X* only, *Z* only, and both *X* and *Z*, respectively, in the study population. (Appendix 1 lists all notations used in this study.) Let Rate_00_(*t*), Rate_10_(*t*), Rate_01_(*t*), and Rate_11_(*t*), represent the respective incidence or mortality rates (depending on the circumstances) for a certain disease in this population at time *t*. Among those people in the study population who contracted the disease (or died of the disease) in a time interval (t,t+Δt) where Δ*t* → 0, the proportions of neither *X* nor *Z* exposure, *X* exposure only, *Z* exposure only, and *X* and *Z* dual exposure can be calculated as $p_{00}^{*}(t)=\frac{p_{00}(t)\times {\text{Rate}}_{00}(t)}{\text{Rate}(t)}$ , $p_{10}^{*}(t)=\frac{p_{10}(t)\times {\text{Rate}}_{10}(t)}{\text{Rate}(t)}$ , $p_{01}^{*}(t)=\frac{p_{01}(t)\times {\text{Rate}}_{01}(t)}{\text{Rate}(t)}$ , and $p_{11}^{*}(t)=\frac{p_{11}(t)\times {\text{Rate}}_{11}(t)}{\text{Rate}(t)}$ , respectively, where Rate(*t*) = *p*_00_(*t*) × Rate_00_(*t*) + *p*_10_(*t*) × Rate_10_(*t*) + *p*_01_(*t*) × Rate_01_(*t*) + *p*_11_(*t*) × Rate_11_(*t*) refers to the incidence or mortality rate at time *t* in the population at large.

**Figure 1.**
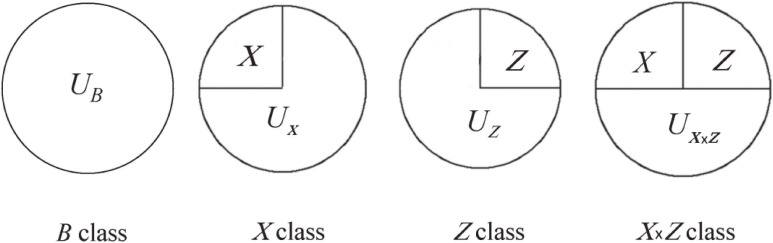
The four classes of causal pies for two binary exposures (*X* and *Z*). The *U* components represent other factors.

To study exposure-disease relationships under the framework of the causal pie model, we invoke Assumption I: sufficient-cause positive monotonicity^7^^,^^8^^,^^19^ (Appendix 2 lists all assumptions invoked in this study). Under the assumption, neither the “absence of *X*”, $\bar{X}$ , nor the “absence of *Z*”, $\bar{Z}$ , can be a component in any class of causal pies and a total of four (rather than nine, if without the assumption) classes of causal pies can be defined for two binary exposures (Figure 1; the *U* components represent factors other than *X* and *Z*). These are the *B* class (ie, the background class, of which neither *X* nor *Z* is a component), the *X* class (of which *X* but not *Z* is a component), the *Z* class (of which *Z* but not *X* is a component), and the *X* × *Z* interaction class (with both *X* and *Z* as components). Let Rate*_B_*(*t*), Rate*_X_*(*t*), Rate*_Z_*(*t*), and Rate*_X_*_×_*_Z_*(*t*) denote the rates at time *t* for the completion of the causal pies of the *B* class, the *X* class, the *Z* class, and the *X* × *Z* interaction class, respectively.

Next, we invoke Assumption II: class-specific completion rates. Under the assumption, a completion rate is specific and only specific to the class of sufficient cause. In other words, the same class of sufficient cause has the same completion rate, irrespective of its possibly differing background factors as well as exposure profiles; the completion rate of the *B* class for any person is Rate*_B_*(*t*) regardless of his/her exposure profile, the completion rate of the *X* class for an *X*-exposed person is Rate*_X_*(*t*) regardless of his/her *Z* status, the completion rate of the *Z* class for a *Z*-exposed person is Rate*_Z_*(*t*) regardless of his/her *X* status, and the completion rate of the *X* × *Z* interaction class is Rate*_X_*_×_*_Z_*(*t*) for a person exposed to both *X* and *Z*.

To relate the two types of rates (completion rate and disease rate), we invoke Assumption III: no redundancy.^20^^,^^21^ The assumption posits that in a sufficiently short time interval (t,t+Δt) where Δ*t* → 0, there can be at most one class of causal pies that can be completed for any individual, and hence, Rate_00_(*t*) = Rate*_B_*(*t*), Rate_10_(*t*) = Rate*_B_*(*t*) + Rate*_X_*(*t*), Rate_01_(*t*) = Rate*_B_*(*t*) + Rate*_Z_*(*t*), and Rate_11_(*t*) = Rate*_B_*(*t*) + Rate*_X_*(*t*) + Rate*_Z_*(*t*) + Rate*_X_*_×_*_Z_*(*t*). Therefore, we have that Rate*_B_*(*t*) = Rate_00_(*t*), Rate*_X_*(*t*) = Rate_10_(*t*) − Rate_00_(*t*), Rate*_Z_*(*t*) = Rate_01_(*t*) − Rate_00_(*t*), and Rate*_X_*_×_*_Z_*(*t*) = Rate_11_(*t*) − Rate_01_(*t*) − Rate_10_(*t*) + Rate_00_(*t*), respectively.^8^^,^^22^

A diseased person exposed to neither *X* nor *Z* can only acquire the disease through the completion of the causal pies of the *B* class (Assumption I), that is, the disease for the person can be entirely attributed to the background causes. For a person exposed to *X* but not *Z*, the probability that the causal pies of the *B* class will complete in a time interval (t,t+Δt) where Δ*t* → 0 is Rate*_B_*(*t*) × Δ*t*, and the corresponding probability for the *X* class is Rate*_X_*(*t*) × Δ*t* (Assumptions I and II). The probability that the person will acquire the disease in (t,t+Δt) is the sum of the two probabilities, Rate*_B_*(*t*) × Δ*t* + Rate*_X_*(*t*) × Δ*t* (Assumption III). If the person indeed acquired the disease in this time interval, the probability that he/she acquired the disease because of the completion of the causal pies of the *B* class is $\frac{{\text{Rate}}_{B}(t)\times \Delta t}{{\text{Rate}}_{B}(t)\times \Delta t+{\text{Rate}}_{X}(t)\times \Delta t}=\frac{{\text{Rate}}_{B}(t)}{{\text{Rate}}_{B}(t)+{\text{Rate}}_{X}(t)}=\frac{{\text{Rate}}_{B}(t)}{{\text{Rate}}_{10}(t)}$ , and that because of the *X* class, $\frac{{\text{Rate}}_{X}(t)\times \Delta t}{{\text{Rate}}_{B}(t)\times \Delta t+{\text{Rate}}_{X}(t)\times \Delta t}=\frac{{\text{Rate}}_{X}(t)}{{\text{Rate}}_{B}(t)+{\text{Rate}}_{X}(t)}=\frac{{\text{Rate}}_{X}(t)}{{\text{Rate}}_{10}(t)}$ . Similarly, the corresponding probabilities for a diseased person only exposed to *Z* are $\frac{{\text{Rate}}_{B}(t)}{{\text{Rate}}_{01}(t)}$ (*B* class) and $\frac{{\text{Rate}}_{Z}(t)}{{\text{Rate}}_{01}(t)}$ (*Z* class), and those for a diseased person exposed to both *X* and *Z*, $\frac{{\text{Rate}}_{B}(t)}{{\text{Rate}}_{11}(t)}$ (*B* class), $\frac{{\text{Rate}}_{X}(t)}{{\text{Rate}}_{11}(t)}$ (*X* class), $\frac{{\text{Rate}}_{Z}(t)}{{\text{Rate}}_{11}(t)}$ (*Z* class), and $\frac{{\text{Rate}}_{X\times Z}(t)}{{\text{Rate}}_{11}(t)}$ (*X* × *Z* interaction class).

We now invoke Assumption IV: stable population. Under the assumption, the proportions of people with various combinations of exposures, the disease rates, and the completion rates in the study population all remain constant over the study period.^1^ Therefore, we can legitimately suppress the dependency on *t*, ie, *p_ij_*(*t*) = *p_ij_*, $p_{ij}^{*}(t)=p_{ij}^{*}$ , and Rate*_ij_*(*t*) = Rate*_ij_* for i,j∈{0,1} , Rate(*t*) = Rate, and Rate*_class_*(*t*) = Rate*_class_* for class∈{B,X,Z,X×Z} in all equations presented above.

### Disease attribution using the causal pie weight

From the above derivations, a diseased person in the study population can be attributed to the following causes using the following apportionment scheme, if Assumptions I–IV hold:

$$\left\{\begin{array}{l} \text{to background causes:}\\ \;{\text{CPW}}_{B}=p_{00}^{*}+p_{10}^{*}\times \frac{{\text{Rate}}_{B}}{{\text{Rate}}_{10}}+p_{01}^{*}\times \frac{{\text{Rate}}_{B}}{{\text{Rate}}_{01}}\\ \;+p_{11}^{*}\times \frac{{\text{Rate}}_{B}}{{\text{Rate}}_{11}}=\frac{{\text{Rate}}_{00}}{\text{Rate}},\\ \text{to }X\text{ exclusively}:\\ \;{\text{CPW}}_{X}=p_{10}^{*}\times \frac{{\text{Rate}}_{X}}{{\text{Rate}}_{10}}+p_{11}^{*}\times \frac{{\text{Rate}}_{X}}{{\text{Rate}}_{11}}\\ \;=(p_{10}+p_{11})\times \frac{{\text{Rate}}_{10}-{\text{Rate}}_{00}}{\text{Rate}},\\ \text{to }Z\text{ exclusively}:\\ \;{\text{CPW}}_{Z}=p_{01}^{*}\times \frac{{\text{Rate}}_{Z}}{{\text{Rate}}_{01}}+p_{11}^{*}\times \frac{{\text{Rate}}_{Z}}{{\text{Rate}}_{11}}\\ \;=(p_{01}+p_{11})\times \frac{{\text{Rate}}_{01}-{\text{Rate}}_{00}}{\text{Rate}},\\ \text{to the interaction between }X\text{ and }Z:\\ \;{\text{CPW}}_{X\times Z}=p_{11}^{*}\times \frac{{\text{Rate}}_{X\times Z}}{{\text{Rate}}_{11}}\\ \;=p_{11}\times \frac{{\text{Rate}}_{11}-{\text{Rate}}_{01}-{\text{Rate}}_{10}+{\text{Rate}}_{00}}{\text{Rate}}, \end{array}\right.$$ (1)

where CPW*_B_*, CPW*_X_*, CPW*_Z_*, and CPW*_X_*_×_*_Z_* are the CPWs for the *B*, *X*, *Z*, and *X* × *Z* interaction classes, respectively.^7^^–^^9^ Arithmetically, the sum of the four CPWs is guaranteed to be 100%. Note that “rates” (rather than “risks” or “odds”) were used here to measure disease occurrences or mortalities.

Essentially, a CPW quantifies the fraction of the disease that can be attributed to a particular class of causal pies: CPW*_B_* for the *B* class or the background causes, CPW*_X_* for the *X* class or the *X* exclusively, CPW*_Z_* for the *Z* class or the *Z* exclusively, and CPW*_X_*_×_*_Z_* for the *X* × *Z* interaction classes or the interaction between *X* and *Z*. The proposed CPW is similar but different to the conventional attributable/etiologic fraction^20^^,^^23^: the former is a measure for a particular class of causal pies, while the latter is for a particular exposure. Notably, using the exposure-specific attributable/etiologic fraction, we can neither attribute the disease to the interaction between exposures, nor to the background causes. From formula (1) above, we see that the weight of the interaction (CPW*_X_*_×_*_Z_*) is nil, if and only if disease occurrences or mortalities conform to an additive model in which the combined effect of *X* and *Z* (in terms of rate difference, Rate_Difference*_X_*_&_*_Z_* = Rate_11_ − Rate_00_) is the sum of the effects that are due to *X* (Rate_Difference*_X_* = Rate_10_ − Rate_00_) and *Z* (Rate_Difference*_Z_* = Rate_01_ − Rate_00_).

To calculate CPWs using formula (1), seven parameters are required: *p*_00_, *p*_10_, *p*_01_ (*p*_11_ = 1 − *p*_00_ − *p*_10_ − *p*_01_), Rate_00_, Rate_10_, Rate_01_, and Rate_11_ (Rate = *p*_00_ × Rate_00_ + *p*_10_ × Rate_10_ + *p*_01_ × Rate_01_ + *p*_11_ × Rate_11_). Alternatively, the three parameters concerning the two exposures can be replaced with the marginal prevalence rate of each (*p_X_* = *p*_10_ + *p*_11_ for *X* and *p_Z_* = *p*_01_ + *p*_11_ for *Z*) and the prevalence odds ratio (OR) between the two ( ${\text{OR}}_{X,Z}=\frac{p_{11}p_{00}}{p_{10}p_{01}}$ ). The four parameters concerning incidence or mortality rates can be replaced with the three rate ratios (Rate_Ratio*_X_* = Rate_10_/Rate_00_, Rate_Ratio*_Z_* = Rate_01_/Rate_00_, and Rate_Ratio*_X_*_&_*_Z_* = Rate_11_/Rate_00_, respectively) and the rate in the population at large.

These parameters can be obtained from multiple data sources, such as the established registries, surveys, and observational studies conducted on the population of interest or other comparable populations, or from literature searches of original studies or meta-analyses on the exposures and the disease of concern. The following conversion formula^24^ can be used if a risk (rather than a rate) is used: $\text{Rate}=\frac{-\log(1-\text{Risk})}{\text{Duration}}$ . The apportionment scheme can be extended to attribute the disease to more than two exposures. For a total of *m* binary exposures, a total of 2*^m^*^+1^ − 1 parameters is required.

If disease occurrences or mortalities conform to a multiplicative model (Rate_Ratio*_X_*_&_*_Z_* = Rate_Ratio*_X_* × Rate_Ratio*_Z_*; Assumption V), and *X* and *Z* are independent in the population [*p*_11_ = *p_X_p_Z_*, *p*_10_ = *p_X_*(1 − *p_Z_*), *p*_01_ = (1 − *p_X_*)*p_Z_*, and *p*_00_ = (1 − *p_X_*)(1 − *p_Z_*); Assumption VI], Appendix 3 shows that CPWs per se also conform to a multiplicative model as follows:

$$\left\{ \begin{array}{l} \text{to background causes:}\\ \;{\text{CPW}}_{B}=(1-{\text{PAF}}_{X})\times (1-{\text{PAF}}_{Z}),\\ \text{to }X\text{ exclusively:}\\ \;{\text{CPW}}_{X}={\text{PAF}}_{X}\times (1-{\text{PAF}}_{Z}),\\ \text{to }Z\text{ exclusively:}\\ \;{\text{CPW}}_{Z}=(1-{\text{PAF}}_{X})\times {\text{PAF}}_{Z},\\ \text{to the interaction between }X\text{ and }Z\text{:}\\ \;{\text{ CPW}}_{X\times Z}={\text{PAF}}_{X}\times {\text{PAF}}_{Z}, \end{array}\right.$$ (2)

where ${\text{PAF}}_{X}=\frac{p_{X}\times ({\text{Rate\_Ratio}}_{X}-1)}{p_{X}\times ({\text{Rate\_Ratio}}_{X}-1)+1}$ and ${\text{PAF}}_{Z}=\frac{p_{\text{Z}}\times ({\text{Rate\_Ratio}}_{Z}-1)}{p_{\text{Z}}\times ({\text{Rate\_Ratio}}_{Z}-1)+1}$ are the marginal PAFs for *X* and *Z*, respectively. Note again that these PAFs are based on “rate ratios” (rather than the usual “risk ratios” or “odds ratios”). To calculate CPWs using formula (2), only two parameters must be input: PAF*_X_* and PAF*_Z_*. For a total of *m* binary exposures (*i* = 1,…,*m*), this apportionment scheme requires only *m* parameters (PAF_1_,…,PAF*_m_*) as in the following formula (Appendix 4):

$${\text{CPW}}_{e_{1},\ldots ,e_{m}}=\prod_{i=1}^{m}(1-{\text{PAF}}_{i}{)}^{1-e_{i}}\times ({\text{PAF}}_{i}{)}^{e_{i}},$$ (3)

where *e_i_* = 1 indicates that the *i*th exposure is present in the causal pie, and that otherwise, *e_i_* = 0.

In the sequel, we use two examples to demonstrate the methodology. The data was taken from papers published in 2010^25^ and 2018^3^ or retrieved from the public domain in 2020.^26^ All analysis was done in 2020.

## RESULTS

### Example 1

To attribute oral cancer mortality in men in Taiwan to cigarette smoking (*X*) and betel quid chewing (*Z*), we extract several summary statistics from a cohort study by Wen et al^25^ as the input parameters to formula (1). These include the exposure-related parameters: *p*_00_ = 46% (the proportion of men in Taiwan who neither smoked nor chewed betel quid), *p*_10_ = 35% (those who only smoked), *p*_01_ = 2% (those who only chewed betel quid), and *p*_11_ = 17% (those who both smoked and chewed betel quid). The disease-related parameters are also included as follows: Rate_Ratio*_X_* = 2.09 (hazard ratio of oral cancer deaths between those who only smoked and those who neither smoked nor chewed betel quid), Rate_Ratio*_Z_* = 3.81 (for those who only chewed betel quid), and Rate_Ratio*_X_*_&_*_Z_* = 9.49 (for those who both smoked and chewed betel quid). We obtain our final input parameter, the mortality rate of oral cancer for men aged ≥20 years in Taiwan in 2000, which was obtained from the Taiwan Cancer Registry Online Interactive Query System (26): Rate = 17.24 per 100,000 persons.

By using the formula (1) (under Assumptions I, II, III, and IV), we conclude that 27.1%, 19.7%, 18.5%, and 34.7% of oral cancer mortality in men in Taiwan is attributable to the interaction between cigarette smoking and betel quid chewing (CPW*_X_*_×_*_Z_* = 0.271), to smoking only (CPW*_X_* = 0.197), to betel quid chewing only (CPW*_Z_* = 0.185), and to neither of the two (CPW*_B_* = 0.347).

Of note, betel quid chewing (Rate_Ratio*_Z_* = 3.81) is a stronger risk factor for oral cancer mortality than is smoking (Rate_Ratio*_X_* = 2.09), but the CPWs for the two are similar (CPW*_Z_* = 0.185 vs CPW*_X_* = 0.197). This is understandable as the prevalence of smoking (*p_X_* = 52%) is higher than that of betel quid chewing (*p_Z_* = 19%). Also notable is a very large CPW for the interaction between cigarette smoking and betel quid chewing (CPW*_X_*_×_*_Z_* = 0.271). This has important public health implications: An areca nut prevention and control program, if it can be successfully implemented in Taiwan to the 19% of people who chewed betel quid, will expect to reduce oral cancer mortality by not just 18.5% but 45.6% (CPW*_Z_* + CPW*_X_*_×_*_Z_* = 0.456).

### Example 2

The GBD 2017 study^3^ quantified the global burden of esophageal cancer mortality for the five major risk factors for the disease using the PAF index: smoking (39.1%), alcohol use (31.8%), high body mass index (18.6%), a diet low in fruits (19.0%), and tobacco chewing (7.3%). The sum of the five PAFs (115.8%) far exceeds the theoretically maximum possible value of 100%, complicating the interpretation of the results. This also leaves no room for other factors that may also contribute to esophageal cancer mortality to share the burden.

We use formula (3) (under Assumptions I, II, …, VI) to reapportion the global burden of esophageal cancer mortality to the risk factors and their interactions (Table 1). The main effects of the five major risk factors account for 41.9% (16.3% + 11.9% + 5.8% + 6.0% + 2.0%) of the global burden of esophageal cancer mortality. The 10 two-factor, 10 three-factor, 5 four-factor, and 1 five-factor interaction account for 25.1%, 6.8%, 0.8%, and 0.03%, respectively. Formula (3) also sets aside 25.4% of the burden for the contribution of other factors.

**Table 1.** Attribution of global esophageal cancer mortality to smoking, alcohol use, high body mass index, a diet low in fruits, tobacco chewing, other risk factors, and the interactions between these factors

Risk Factors or Interactions^a^ Between Two Factors	Causal Pie Weight (%)	Interactions^a^ Between More Than Two Factors	Causal Pie Weight (%)
	
O^b^	25.37	S × A × B	1.74
S^c^	16.28	S × A × D	1.79
A^d^	11.85	S × A × T	0.60
B^e^	5.80	S × B × D	0.87
D^f^	5.96	S × B × T	0.29
T^g^	2.00	S × D × T	0.30
S × A	7.61	A × B × D	0.64
S × B	3.72	A × B × T	0.21
S × D	3.82	A × D × T	0.22
S × T	1.28	B × D × T	0.11
A × B	2.71	S × A × B × D	0.41
A × D	2.78	S × A × B × T	0.14
A × T	0.93	S × A × D × T	0.14
B × D	1.36	S × B × D × T	0.07
B × T	0.46	A × B × D × T	0.05
D × T	0.47	S × A × B × D × T	0.03

^a^Denoted as “×”.

^b^Other risk factors.

^c^Smoking.

^d^Alcohol use.

^e^High body mass index.

^f^A diet low in fruits.

^g^Tobacco chewing.

## DISCUSSION

In this paper, we propose a disease attribution method using aggregate data, possibly from multiple data sources, under which the burden of disease is apportioned to the independent and the interaction effects of each of its major risk factors as well as all the other factors as a whole. This apportionment scheme guarantees the total share of the burden to be 100%. As mentioned, this paper is based on the causal pie model.^1^^,^^7^^–^^9^^,^^18^ To use formula (1) for disease attribution, we need a total of four assumptions: (I) sufficient-cause positive monotonicity, (II) class-specific completion rates, (III) no redundancy, and (IV) stable population. To use formulas (2) and (3), we need two more assumptions: (V) multiplicative model, and (VI) independent exposures. All these assumptions are difficult to check.

Assumption I is stronger than the “counterfactual positive monotonicity assumption”.^19^ One may argue that either assumption likely holds given the implausibility of carcinogens preventing cancer. But this is not always the case; even well-recognized risk factors can have some beneficial effects, such as light to moderate alcohol consumption, in reducing the risk of cardiovascular diseases. Assumption II cannot be guaranteed, even in ideal randomized controlled trials. One may need to perform subgroup analysis by conditioning on factors other than the exposures under study, hoping that the assumption will hold at least approximately in the more homogeneous population so defined. Assumption III is a Poisson-like assumption. The assumption will fail when the unknown complement causes for two different classes of causal pies share a common component that happens to be the last one to complete among all of the unknown component causes of these two classes before all other classes are completed. Assumption IV is a strong assumption, given the changing nature of most exposures and diseases. But the assumption is reasonable or approximately so when the follow-up time is not too long (for example, less than 5 years). In situations when Assumption V or VI fails, one should use the more demanding formula (1) for disease attribution. If these assumptions hold or are approximately so, one can then use the more convenient formula (2) or (3) which only requires the data of exposure-specific PAFs. Finally, if data are taken from multiple sources, we need to ensure that they share similar characteristics, such as sex, age, race, exposure prevalence, and disease rate. The two examples are presented in this paper merely to demonstrate the methodology.

In this paper, we use rate rather than risk to measure disease occurrence or mortality for the following reasons. First, because the risk is dependent on follow-up duration, two researchers using the measure may attribute the disease of concern to the same exposure differently simply because they use data from epidemiological studies with different follow-up durations. Second, a risk increases (and eventually approaches 1 if it is all-cause mortality risk) as the follow-up duration increases. Therefore, the fraction of a disease that can be attributable to any exposure may become vanishingly small over time. For example, both smokers and nonsmokers will eventually die, and if their risks of death are compared, death attributable to smoking becomes 0. By contrast, a rate is independent of follow-up duration. Over time, a risk ratio (of death) between the exposed and unexposed people will approach 1 but a rate ratio will not, and a PAF based on the risk ratio will approach 0 but a PAF based on the rate ratio will not. Finally, a rate is a risk divided by a time interval when the time interval approaches zero. It is in such an infinitely small time interval that we are in a position to impose the no redundancy assumption and greatly simplify the total number of (time-dependent) response types^27^ in the potential outcome (counterfactual) model for risk.

Several issues warrant further investigations. First, controlling for confounding is essential for causal inference in observational studies. A simple subgroup analysis will be sufficient for a binary confounder, such as sex. However, standardization and/or regression methods need to be developed to deal with a confounder on a polytomous (such as age groups) or continuous (such as personal income) scale, and the situations where many confounders need to be conditioned upon simultaneously. Second, statistical inference procedures also need to be developed to allow for hypothesis testing and interval estimation regarding the CPW indices, especially since the statistics are from aggregate data, and possibly also from different sources. Third, as pointed out, the method relies on many assumptions. Sensitivity analysis procedures, therefore, need to be developed to check the robustness of the proposed method to these assumptions. Fourth, further studies are warranted to incorporate the CPW indices into a cost-effectiveness analysis to inform policy. Finally, the causal pie model upon which our method is based specifies which exposures are participating in a particular interaction class of causal pies but not their relative contributions. If disease attribution to exposures is the desired end, how the participating exposures share the weight of the interaction class needs further study.

In summary, we propose a method for disease attribution to multiple exposures using aggregate data. The calculation is simple and straightforward; therefore, we recommend its use for studies on disease burden.

## SUPPLEMENTARY MATERIAL

Appendix 1. Notations used in the study

Appendix 2. Assumptions invoked in the study

Appendix 3. Derivations of Equation (2) in the study

Appendix 4. Derivations of Equation (3) in the study
