# Supplementary material for: Disease Attribution to Multiple Exposures Using Aggregate Data
Source: J Epidemiol. 2023 Aug 5;33(8):405–9. doi: 10.2188/jea.JE20210084 (PMC10319529; doi:10.2188/jea.JE20210084)
Supplement: Supplementary file 1 [file je-33-405-s001.pdf]

Appendix 1. Notations used in the study

$p_{00}(t)$, $p_{10}(t)$, $p_{01}(t)$, and $p_{11}(t)$:

proportions at time *t* of people exposed to neither *X* nor *Z*, *X* only, *Z* only, and both *X* and *Z*, respectively, in the study population; the numerators are the numbers at time *t* of people exposed to neither *X* nor *Z*, *X* only, *Z* only, and both *X* and *Z*, respectively, in the study population, and the (common) denominator for all these proportions is the total number of people at time *t* in the study population. Note that these are the exposure prevalence *at time t*, not the exposure risks (the risks of being exposed) *by time t.*

$\text{Rate}_{00}(t)$, $\text{Rate}_{10}(t)$, $\text{Rate}_{01}(t)$, and $\text{Rate}_{11}(t)$:

incidence or mortality rates for a certain disease at time t of people exposed to neither *X* nor *Z*, *X* only, *Z* only, and both *X* and *Z*, respectively, in the study population.

$\text{Rate}(t)$:

incidence or mortality rate at time *t* in the population at large;

$\text{Rate}(t) = p_{00}(t)\times\text{Rate}_{00}(t) + p_{10}(t)\times\text{Rate}_{10}(t) + p_{01}(t)\times\text{Rate}_{01}(t) + p_{11}(t)\times\text{Rate}_{11}(t)$.

$p^*_{00}(t)$, $p^*_{10}(t)$, $p^*_{01}(t)$, and $p^*_{11}(t)$:

proportions of neither *X* nor *Z* exposure, *X* exposure only, *Z* exposure only, and *X* and *Z* dual exposure, respectively, among those people in the study population who contracted the disease (or died of the disease) in a time interval $(t,\ t+\triangle t)$ where $\triangle t \to 0$; $p^*_{00}(t) = \dfrac{p_{00}(t)\times\text{Rate}_{00}(t)}{\text{Rate}(t)}$, $p^*_{10}(t) = \dfrac{p_{10}(t)\times\text{Rate}_{10}(t)}{\text{Rate}(t)}$,

$p^*_{01}(t) = \dfrac{p_{01}(t)\times\text{Rate}_{01}(t)}{\text{Rate}(t)}$, and $p^*_{11}(t) = \dfrac{p_{11}(t)\times\text{Rate}_{11}(t)}{\text{Rate}(t)}$, respectively.

$\text{Rate}_B(t)$, $\text{Rate}_X(t)$, $\text{Rate}_Z(t)$, and $\text{Rate}_{X\times Z}(t)$:

completion rates at time *t* of the causal pies of the *B* class, the *X* class, the *Z* class, and the $X\times Z$ interaction class, respectively.

Under Assumptions I, II and III, $\text{Rate}_B(t) = \text{Rate}_{00}(t)$, $\text{Rate}_X(t) = \text{Rate}_{10}(t) - \text{Rate}_{00}(t)$,

$\text{Rate}_Z(t) = \text{Rate}_{01}(t) - \text{Rate}_{00}(t)$, and $\text{Rate}_{X\times Z}(t) = \text{Rate}_{11}(t) - \text{Rate}_{01}(t) - \text{Rate}_{10}(t) + \text{Rate}_{00}(t)$,

respectively.

$\text{Rate\_Difference}_X(t)$:

rate difference at time *t*; $\text{Rate}_{10}(t) - \text{Rate}_{00}(t)$.

$\text{Rate\_Difference}_Z(t)$:

rate difference at time *t*; $\text{Rate}_{01}(t) - \text{Rate}_{00}(t)$.

$\text{Rate\_Difference}_{X\&Z}(t)$:

rate difference at time *t*; $\text{Rate}_{11}(t) - \text{Rate}_{00}(t)$.

$p_X(t)$ and $p_Z(t)$:

marginal prevalence rates at time *t* for *X* and *Z*, respectively; $p_X(t) = p_{10}(t) + p_{11}(t)$ and

$p_Z(t) = p_{01}(t) + p_{11}(t)$, respectively.

$\text{OR}_{X,Z}(t)$:

prevalence odds ratio at time *t* between *X* and *Z*; $\text{OR}_{X,Z}(t) = \dfrac{p_{11}(t)\,p_{00}(t)}{p_{10}(t)\,p_{01}(t)}$.

$\text{Rate\_Ratio}_X(t)$:

rate ratio at time *t*; $\text{Rate}_{10}(t)/\text{Rate}_{00}(t)$.

$\text{Rate\_Ratio}_Z(t)$:

rate ratio at time *t*; $\text{Rate}_{01}(t)/\text{Rate}_{00}(t)$.

$\text{Rate\_Ratio}_{X\&Z}(t)$:

rate ratio at time *t*; $\text{Rate}_{11}(t)/\text{Rate}_{00}(t)$.

$\text{PAF}_X(t)$ and $\text{PAF}_Z(t)$:

marginal population attributable fractions at time t for X and Z, respectively;

$\mathrm{PAF}_X(t) = \dfrac{p_X(t)\times[\mathrm{Rate\_Ratio}_X(t)-1]}{p_X(t)\times[\mathrm{Rate\_Ratio}_X(t)-1]+1}$ and $\mathrm{PAF}_Z = \dfrac{p_Z\times[\mathrm{Rate\_Ratio}_Z-1]}{p_Z\times[\mathrm{Rate\_Ratio}_Z-1]+1}$, respectively.

$\mathrm{CPW}_B(t)$, $\mathrm{CPW}_X(t)$, $\mathrm{CPW}_Z(t)$, and $\mathrm{CPW}_{X\times Z}(t)$:

causal-pie weights at time t for the B class, the X class, the Z class, and the $X\times Z$ interaction class, respectively.

Under Assumptions I, II, and III, $\mathrm{CPW}_B(t) = \dfrac{\mathrm{Rate}_{00}(t)}{\mathrm{Rate}(t)}$, $\mathrm{CPW}_X(t) = [p_{10}(t)+p_{11}(t)]\times\dfrac{\mathrm{Rate}_{10}(t)-\mathrm{Rate}_{00}(t)}{\mathrm{Rate}(t)}$,

$\mathrm{CPW}_Z(t) = [p_{01}(t)+p_{11}(t)]\times\dfrac{\mathrm{Rate}_{01}(t)-\mathrm{Rate}_{00}(t)}{\mathrm{Rate}(t)}$, and

$\mathrm{CPW}_{X\times Z}(t) = p_{11}(t)\times\dfrac{\mathrm{Rate}_{11}(t)-\mathrm{Rate}_{01}(t)-\mathrm{Rate}_{10}(t)+\mathrm{Rate}_{00}(t)}{\mathrm{Rate}(t)}$, respectively.

Under Assumptions I, II, III, V and VI, $\mathrm{CPW}_B(t) = [1-\mathrm{PAF}_X(t)]\times[1-\mathrm{PAF}_Z(t)]$,

$\mathrm{CPW}_X(t) = \mathrm{PAF}_X(t)\times[1-\mathrm{PAF}_Z(t)]$, $\mathrm{CPW}_Z(t) = [1-\mathrm{PAF}_X(t)]\times\mathrm{PAF}_Z(t)$, and

$\mathrm{CPW}_{X\times Z}(t) = \mathrm{PAF}_X(t)\times\mathrm{PAF}_Z(t)$, respectively.

$\mathrm{PAF}_1(t),\ldots,\mathrm{PAF}_m(t)$:

marginal population attributable fractions at time t for a total of m binary exposures.

$\mathrm{CPW}_{e_1,\ldots,e_m}(t)$:

causal-pie weight at time t for a particular class of causal pies, where $e_i = 1$ indicates that the i th exposure is present, and that otherwise, $e_i = 0$.

Under Assumptions I, II, III, V, and VI, $\mathrm{CPW}_{e_1,\ldots,e_m}(t) = \prod_{i=1}^{m}[1-\mathrm{PAF}_i(t)]^{1-e_i}\times[\mathrm{PAF}_i(t)]^{e_i}$.

p_{00}, p_{10}, p_{01}, and p_{11}:

proportions at any time t within the study period of people exposed to neither X nor Z, X only, Z only, and both X and Z, respectively, in the study population under Assumption IV; the numerators are the numbers at

time *t* of people exposed to neither *X* nor *Z*, *X* only, *Z* only, and both *X* and *Z*, respectively, in the study population, and the (common) denominator for all these proportions is the total number of people at time *t* in the study population. Note that these are the exposure prevalence *at time t*, not the exposure risks (the risks of being exposed) *by time t.*

Rate_{00}, Rate_{10}, Rate_{01}, and Rate_{11}:

incidence or mortality rates for a certain disease at any time *t* within the study period of people exposed to neither *X* nor *Z*, *X* only, *Z* only, and both *X* and *Z*, respectively, in the study population under Assumption IV.

Rate:

incidence or mortality rate at any time *t* within the study period in the population at large under Assumption IV; $\text{Rate} = p_{00} \times \text{Rate}_{00} + p_{10} \times \text{Rate}_{10} + p_{01} \times \text{Rate}_{01} + p_{11} \times \text{Rate}_{11}$.

p^*_{00}, p^*_{10}, p^*_{01}, and p^*_{11}:

proportions of neither *X* nor *Z* exposure, *X* exposure only, *Z* exposure only, and *X* and *Z* dual exposure, respectively, among those people in the study population who contracted the disease (or died of the disease) at any time *t* within the study period under Assumption IV; $p^*_{00} = \frac{p_{00} \times \text{Rate}_{00}}{\text{Rate}}$, $p^*_{10} = \frac{p_{10} \times \text{Rate}_{10}}{\text{Rate}}$, $p^*_{01} = \frac{p_{01} \times \text{Rate}_{01}}{\text{Rate}}$, and $p^*_{11} = \frac{p_{11} \times \text{Rate}_{11}}{\text{Rate}}$, respectively.

Rate_B, Rate_X, Rate_Z, and $\text{Rate}_{X \times Z}$:

completion rates at any time *t* within the study period of the causal pies of the *B* class, the *X* class, the *Z* class, and the $X \times Z$ interaction class, respectively, under Assumption IV.

If together with Assumptions I, II and III, $\text{Rate}_B = \text{Rate}_{00}$, $\text{Rate}_X = \text{Rate}_{10} - \text{Rate}_{00}$,

$\text{Rate}_Z = \text{Rate}_{01} - \text{Rate}_{00}$, and $\text{Rate}_{X \times Z} = \text{Rate}_{11} - \text{Rate}_{01} - \text{Rate}_{10} + \text{Rate}_{00}$, respectively.

Rate_Difference_X:

rate difference at any time *t* within the study period under Assumption IV; $\text{Rate}_{10} - \text{Rate}_{00}$.

Rate_Difference_Z :

rate difference at any time *t* within the study period under Assumption IV; $\text{Rate}_{01} - \text{Rate}_{00}$.

$\text{Rate\_Difference}_{X\&Z}$:

rate difference at any time *t* within the study period under Assumption IV; $\text{Rate}_{11} - \text{Rate}_{00}$.

p_X and p_Z :

marginal prevalence rates at any time *t* within the study period for *X* and *Z*, respectively, under Assumption IV; $p_X = p_{10} + p_{11}$ and $p_Z = p_{01} + p_{11}$, respectively.

$\text{OR}_{X,Z}$:

prevalence odds ratio at any time *t* within the study period between *X* and *Z* under Assumption IV;

$$\text{OR}_{X,Z} = \frac{p_{11} p_{00}}{p_{10} p_{01}} .$$

Rate_Ratio_X :

rate ratio at any time *t* within the study period under Assumption IV; $\text{Rate}_{10} / \text{Rate}_{00}$.

Rate_Ratio_Z :

rate ratio at any time *t* within the study period under Assumption IV; $\text{Rate}_{01} / \text{Rate}_{00}$.

$\text{Rate\_Ratio}_{X\&Z}$:

rate ratio at any time *t* within the study period under Assumption IV; $\text{Rate}_{11} / \text{Rate}_{00}$.

PAF_X and PAF_Z :

marginal population attributable fractions at any time *t* within the study period for *X* and *Z*, respectively, under Assumption IV; $\text{PAF}_X = \frac{p_X \times (\text{Rate\_Ratio}_X - 1)}{p_X \times (\text{Rate\_Ratio}_X - 1) + 1}$ and $\text{PAF}_Z = \frac{p_Z \times (\text{Rate\_Ratio}_Z - 1)}{p_Z \times (\text{Rate\_Ratio}_Z - 1) + 1}$,

respectively.

CPW_B , CPW_X , CPW_Z , and $\text{CPW}_{X\times Z}$:

causal-pie weights at any time *t* within the study period for the *B* class, the *X* class, the *Z* class, and the $X \times Z$ interaction class, respectively, under Assumption IV.

If together with Assumptions I, II, and III, $\text{CPW}_B = \frac{\text{Rate}_{00}}{\text{Rate}}$, $\text{CPW}_X = (p_{10} + p_{11}) \times \frac{\text{Rate}_{10} - \text{Rate}_{00}}{\text{Rate}}$,

$\text{CPW}_Z = (p_{01} + p_{11}) \times \frac{\text{Rate}_{01} - \text{Rate}_{00}}{\text{Rate}}$, and $\text{CPW}_{X \times Z} = p_{11} \times \frac{\text{Rate}_{11} - \text{Rate}_{01} - \text{Rate}_{10} + \text{Rate}_{00}}{\text{Rate}}$,

respectively.

If together with Assumptions I, II, III, V and VI, $\text{CPW}_B = (1 - \text{PAF}_X) \times (1 - \text{PAF}_Z)$,

$\text{CPW}_X = \text{PAF}_X \times (1 - \text{PAF}_Z)$, $\text{CPW}_Z = (1 - \text{PAF}_X) \times \text{PAF}_Z$, and $\text{CPW}_{X \times Z} = \text{PAF}_X \times \text{PAF}_Z$,

respectively.

$\text{PAF}_1, \ldots, \text{PAF}_m$:

marginal population attributable fractions at any time *t* within the study period for a total of *m* binary exposures under Assumption IV.

$\text{CPW}_{e_1, \ldots, e_m}$:

causal-pie weight at any time *t* within the study period for a particular class of causal pies under Assumption IV, where $e_i = 1$ indicates that the i th exposure is present, and that otherwise, $e_i = 0$.

If together with Assumptions I, II, III, V, and VI, $\text{CPW}_{e_1, \ldots, e_m} = \prod_{i=1}^{m} (1 - \text{PAF}_i)^{1-e_i} \times (\text{PAF}_i)^{e_i}$.

Appendix 2. Assumptions invoked in the study

(I) sufficient-cause positive monotonicity:

Under the assumption, neither the "absence of *X*" nor the "absence of *Z*" can be a component in any class of causal pies and a total of four classes of causal pies can be defined for two binary exposures as shown below, where *X* and *Z* indicates the "presence of *X*" and the "presence of *Z*", respectively:

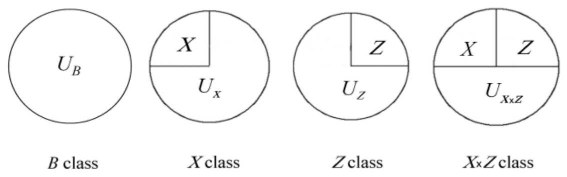

(II) class-specific completion rates:

Under the assumption, a completion rate is specific and only specific to the class of sufficient cause. In other words, the same class of sufficient cause has the same completion rate, irrespective of its possibly differing background factors as well as exposure profiles:

completion rate at time *t* of the *B* class for people exposed to neither *X* nor *Z*: $\text{Rate}_B(t)$,

completion rate at time *t* of the *B* class for people exposed to *X* only: $\text{Rate}_B(t)$,

completion rate at time *t* of the *B* class for people exposed to *Z* only: $\text{Rate}_B(t)$,

completion rate at time *t* of the *B* class for people exposed to both *X* and *Z*: $\text{Rate}_B(t)$,

completion rate at time *t* of the *X* class for people exposed to *X* only: $\text{Rate}_X(t)$,

completion rate at time *t* of the *X* class for people exposed to both *X* and *Z*: $\text{Rate}_X(t)$,

completion rate at time *t* of the *Z* class for people exposed to *Z* only: $\text{Rate}_Z(t)$,

completion rate at time *t* of the *Z* class for people exposed to both *X* and *Z*: $\text{Rate}_Z(t)$,

and

completion rate at time t of the $X \times Z$ interaction class for people exposed to both X and Z: $\text{Rate}_{X\times Z}(t)$.

(III) no redundancy:

Under the assumption, there can be at most one class of causal pies that can be completed for any individual in a sufficiently short time interval:

$$\lim_{\Delta t \to 0} \Pr\left(N_{(t,t+\Delta t)} \geq 2\right) = 0 \quad \text{for any} \quad t,$$

where $N_{(t,t+\Delta t)}$ is a random variable counting the number of causal pies completed in the interval $(t,\ t+\Delta t)$ for a person.

(IV) stable population:

Under the assumption, the proportions of people with various combinations of exposures, the disease rates, and the completion rates in the study population all remain constant over the study period:

$$p_{00}(t) = p_{00},$$

$$p_{10}(t) = p_{10},$$

$$p_{01}(t) = p_{01},$$

$$p_{11}(t) = p_{11},$$

$$p_{00}^{*}(t) = p_{00}^{*},$$

$$p_{10}^{*}(t) = p_{10}^{*},$$

$$p_{01}^{*}(t) = p_{01}^{*},$$

$$p_{11}^{*}(t) = p_{11}^{*},$$

$$\text{Rate}_{00}(t) = \text{Rate}_{00},$$

$$\text{Rate}_{10}(t) = \text{Rate}_{10},$$

$$\text{Rate}_{01}(t) = \text{Rate}_{01},$$

$$\text{Rate}_{11}(t) = \text{Rate}_{11},$$

$$\text{Rate}(t) = \text{Rate},$$

$$\text{Rate}_B(t) = \text{Rate}_B,$$

$$\text{Rate}_X(t) = \text{Rate}_X,$$

$$\text{Rate}_Z(t) = \text{Rate}_Z,$$

and

$$\text{Rate}_{X\times Z}(t) = \text{Rate}_{X\times Z}.$$

(V) multiplicative model:

Under the assumption, we have that

$$\text{Rate\_Ratio}_{X\&Z}(t) = \text{Rate\_Ratio}_X(t) \times \text{Rate\_Ratio}_Z(t),$$

and if together with Assumption IV,

$$\text{Rate\_Ratio}_{X\&Z} = \text{Rate\_Ratio}_X \times \text{Rate\_Ratio}_Z.$$

(VI) independent exposures:

Under the assumption, we have that

$$p_{11}(t) = p_X(t)p_Z(t), \quad p_{10}(t) = p_X(t)\left[1-p_Z(t)\right], \quad p_{01}(t) = \left[1-p_X(t)\right]p_Z(t), \text{ and}$$

$$p_{00}(t) = \left[1-p_X(t)\right]\left[1-p_Z(t)\right],$$

and if together with Assumption IV,

$$p_{11} = p_X p_Z, \quad p_{10} = p_X(1-p_Z), \quad p_{01} = (1-p_X)p_Z, \text{ and } \quad p_{00} = (1-p_X)(1-p_Z).$$

Appendix 3. Derivations of Equation (2) in the study

Under Assumption IV, we have that

$$\begin{aligned}
\frac{\text{Rate}}{\text{Rate}_{00}} &= \frac{p_{11}\times\text{Rate}_{11} + p_{10}\times\text{Rate}_{10} + p_{01}\times\text{Rate}_{01} + p_{00}\times\text{Rate}_{00}}{\text{Rate}_{00}} \\
&= p_{11}\times\text{Rate_Ratio}_{X\&Z} + p_{10}\times\text{Rate_Ratio}_{X} + p_{01}\times\text{Rate_Ratio}_{Z} + p_{00} \\
&= p_X\times p_Z\times\text{Rate_Ratio}_X\times\text{Rate_Ratio}_Z + p_X\times(1-p_Z)\times\text{Rate_Ratio}_X \\
&\quad +(1-p_X)\times p_Z\times\text{Rate_Ratio}_Z+(1-p_X)\times(1-p_Z) \quad \text{(because of Assumptions V and VI)} \\
&=\left[p_X\times\text{Rate_Ratio}_X+(1-p_X)\right]\times\left[p_Z\times\text{Rate_Ratio}_Z+(1-p_Z)\right],
\end{aligned}$$

$$\text{PAF}_X = \frac{p_X\times\left(\text{Rate\_Ratio}_X-1\right)}{p_X\times\left(\text{Rate\_Ratio}_X-1\right)+1} = \frac{p_X\times\left(\text{Rate\_Ratio}_X-1\right)}{p_X\times\text{Rate\_Ratio}_X+\left(1-p_X\right)},$$

$$1-\text{PAF}_X = 1-\frac{p_X\times\left(\text{Rate\_Ratio}_X-1\right)}{p_X\times\text{Rate\_Ratio}_X+\left(1-p_X\right)} = \frac{1}{p_X\times\text{Rate\_Ratio}_X+\left(1-p_X\right)},$$

$$\text{PAF}_Z = \frac{p_Z\times\left(\text{Rate\_Ratio}_Z-1\right)}{p_Z\times\left(\text{Rate\_Ratio}_Z-1\right)+1} = \frac{p_Z\times\left(\text{Rate\_Ratio}_Z-1\right)}{p_Z\times\text{Rate\_Ratio}_Z+\left(1-p_Z\right)},$$

and

$$1-\text{PAF}_Z = 1-\frac{p_Z\times\left(\text{Rate\_Ratio}_Z-1\right)}{p_Z\times\text{Rate\_Ratio}_Z+\left(1-p_Z\right)} = \frac{1}{p_Z\times\text{Rate\_Ratio}_Z+\left(1-p_Z\right)}.$$

Therefore, we have that

$$\mathrm{CPW}_B = \frac{\mathrm{Rate}_{00}}{\mathrm{Rate}} \quad \text{(because of Assumptions I, II, and III)}$$

$$= \frac{1}{\left[p_X \times \mathrm{Rate\_Ratio}_X + (1-p_X)\right] \times \left[p_Z \times \mathrm{Rate\_Ratio}_Z + (1-p_Z)\right]} = (1-\mathrm{PAF}_X) \times (1-\mathrm{PAF}_Z),$$

$$\mathrm{CPW}_X = (p_{10} + p_{11}) \times \frac{\mathrm{Rate}_{10} - \mathrm{Rate}_{00}}{\mathrm{Rate}} \quad \text{(because of Assumptions I, II, and III)}$$

$$= \left[p_X \times (1-p_Z) + p_X \times p_Z\right] \times (\mathrm{Rate\_Ratio}_X - 1) \times \frac{\mathrm{Rate}_{00}}{\mathrm{Rate}} \quad \text{(because of Assumption VI)}$$

$$= \frac{p_X \times (\mathrm{Rate\_Ratio}_X - 1) \times 1}{\left[p_X \times \mathrm{Rate\_Ratio}_X + (1-p_X)\right] \times \left[p_Z \times \mathrm{Rate\_Ratio}_Z + (1-p_Z)\right]} = \mathrm{PAF}_X \times (1-\mathrm{PAF}_Z),$$

$$\mathrm{CPW}_Z = (p_{01} + p_{11}) \times \frac{\mathrm{Rate}_{01} - \mathrm{Rate}_{00}}{\mathrm{Rate}} \quad \text{(because of Assumptions I, II, and III)}$$

$$= \left[(1-p_X) \times p_Z + p_X \times p_Z\right] \times (\mathrm{Rate\_Ratio}_Z - 1) \times \frac{\mathrm{Rate}_{00}}{\mathrm{Rate}} \quad \text{(because of Assumption VI)}$$

$$= \frac{1 \times p_Z \times (\mathrm{Rate\_Ratio}_Z - 1)}{\left[p_X \times \mathrm{Rate\_Ratio}_X + (1-p_X)\right] \times \left[p_Z \times \mathrm{Rate\_Ratio}_Z + (1-p_Z)\right]} = (1-\mathrm{PAF}_X) \times \mathrm{PAF}_Z,$$

and

$$\mathrm{CPW}_{X \times Z} = p_{11} \times \frac{\mathrm{Rate}_{11} - \mathrm{Rate}_{01} - \mathrm{Rate}_{10} + \mathrm{Rate}_{00}}{\mathrm{Rate}} \quad \text{(because of Assumptions I, II, and III)}$$

$$= p_{11} \times (\mathrm{Rate\_Ratio}_{X\&Z} - \mathrm{Rate\_Ratio}_Z - \mathrm{Rate\_Ratio}_X + 1) \times \frac{\mathrm{Rate}_{00}}{\mathrm{Rate}}$$

$$= (p_X \times p_Z) \times (\mathrm{Rate\_Ratio}_X \times \mathrm{Rate\_Ratio}_Z - \mathrm{Rate\_Ratio}_Z - \mathrm{Rate\_Ratio}_X + 1) \times \frac{\mathrm{Rate}_{00}}{\mathrm{Rate}} \quad \text{(because of Assumptions V and VI)}$$

$$= \frac{p_X \times (\mathrm{Rate\_Ratio}_X - 1) \times p_Z \times (\mathrm{Rate\_Ratio}_Z - 1)}{\left[p_X \times \mathrm{Rate\_Ratio}_X + (1-p_X)\right] \times \left[p_Z \times \mathrm{Rate\_Ratio}_Z + (1-p_Z)\right]} = \mathrm{PAF}_X \times \mathrm{PAF}_Z.$$

Appendix 4. Derivations of Equation (3) in the study

With only one exposure, X_1, we have that (under Assumption IV) $p_0^* = \frac{p_0 \times \text{Rate}_0}{\text{Rate}}$ and $p_1^* = \frac{p_1 \times \text{Rate}_1}{\text{Rate}}$, where $\text{Rate} = p_1 \times \text{Rate}_1 + p_0 \times \text{Rate}_0$. We also have that (under Assumptions I, II, and III) $\text{Rate}_0 = \text{Rate}_B$ and $\text{Rate}_1 = \text{Rate}_B + \text{Rate}_{X_1}$, and therefore, $\text{Rate}_B = \text{Rate}_0$ and $\text{Rate}_{X_1} = \text{Rate}_1 - \text{Rate}_0$. A diseased person not exposed to X_1 can be entirely attributed to the *B* class of causal pies, whereas a diseased person exposed to X_1 is attributable to the *B* class of causal pies with a probability of $\frac{\text{Rate}_B}{\text{Rate}_1}$, and to the X_1 class, a probability of $\frac{\text{Rate}_{X_1}}{\text{Rate}_1}$. Therefore, in the study population, the weight for the class of causal pies not containing the X_1 as a component is

$$\begin{aligned}
\text{CPW}_0 &= p_0^* + p_1^* \times \frac{\text{Rate}_B}{\text{Rate}_1} \\
&= \frac{p_0 \times \text{Rate}_0}{\text{Rate}} + \frac{p_1 \times \text{Rate}_1}{\text{Rate}} \times \frac{\text{Rate}_B}{\text{Rate}_1} \\
&= \frac{p_0 \times \text{Rate}_0 + p_1 \times \text{Rate}_B}{\text{Rate}} \\
&= \frac{p_0 \times \text{Rate}_0 + p_1 \times \text{Rate}_0}{p_1 \times \text{Rate}_1 + p_0 \times \text{Rate}_0} \\
&= \frac{1}{p_1 \times \text{Rate_Ratio}_1 + (1 - p_1)} \\
&= 1 - \frac{p_1 \times (\text{Rate_Ratio}_1 - 1)}{p_1 \times (\text{Rate_Ratio}_1 - 1) + 1} \\
&= 1 - \text{PAF}_1
\end{aligned}$$

and the weight for the class of causal pies containing the X_1 as a component is

$$
\begin{aligned}
\text{CPW}_1 &= p_1^* \times \frac{\text{Rate}_{X_1}}{\text{Rate}_1} \\
&= \frac{p_1 \times \text{Rate}_1}{\text{Rate}} \times \frac{\text{Rate}_{X_1}}{\text{Rate}_1} \\
&= \frac{p_1 \times \text{Rate}_{X_1}}{\text{Rate}} \\
&= \frac{p_1 \times \left(\text{Rate}_1 - \text{Rate}_0\right)}{p_1 \times \text{Rate}_1 + p_0 \times \text{Rate}_0} \\
&= \frac{p_1 \times \left(\text{Rate\_Ratio}_1 - 1\right)}{p_1 \times \text{Rate\_Ratio}_1 + (1 - p_1)} \\
&= \frac{p_1 \times \left(\text{Rate\_Ratio}_1 - 1\right)}{p_1 \times \left(\text{Rate\_Ratio}_1 - 1\right) + 1} \\
&= \text{PAF}_1 .
\end{aligned}
$$

This can be more succinctly expressed as

$$
\begin{aligned}
\text{CPW}_{e_1} &= \left(1 - \text{PAF}_1\right)^{1-e_1} \times \left(\text{PAF}_1\right)^{e_1} \\
&= \prod_{i=1}^{1} \left(1 - \text{PAF}_i\right)^{1-e_i} \times \left(\text{PAF}_i\right)^{e_i} ,
\end{aligned}
$$

where $e_1 = 1$ indicates the presence of X_1 in the causal pie, and $e_1 = 0$, otherwise.

With two exposures, X_1 and X_2, Appendix 3 shows that

$$\text{CPW}_{00} = \left(1 - \text{PAF}_1\right) \times \left(1 - \text{PAF}_2\right) ,$$

$$\text{CPW}_{10} = \text{PAF}_1 \times \left(1 - \text{PAF}_2\right) ,$$

$$\text{CPW}_{01} = \left(1 - \text{PAF}_1\right) \times \text{PAF}_2 ,$$

and

$$\text{CPW}_{11} = \text{PAF}_1 \times \text{PAF}_2 .$$

Treating X_1 as the existing factor, and X_2, the add-on factor, we can cast the above equations as

$$\begin{aligned}\mathrm{CPW}_{e_1,e_2} &= [\text{Re the existing factor}]\times[\text{Re the add-on factor}] \\ &= \left[\prod_{i=1}^{1}(1-\mathrm{PAF}_i)^{1-e_i}\times(\mathrm{PAF}_i)^{e_i}\right]\times\left[(1-\mathrm{PAF}_2)^{1-e_2}\times(\mathrm{PAF}_2)^{e_2}\right] \\ &= \prod_{i=1}^{2}(1-\mathrm{PAF}_i)^{1-e_i}\times(\mathrm{PAF}_i)^{e_i}.\end{aligned}$$

With three exposures, X_1, X_2, and X_3, we can treat X_1 and X_2 as the existing factors, and X_3, as the add-on factor. Therefore,

$$\begin{aligned}\mathrm{CPW}_{e_1,e_2,e_3} &= [\text{Re the two existing factors}]\times[\text{Re the add-on factor}] \\ &= \left[\prod_{i=1}^{2}(1-\mathrm{PAF}_i)^{1-e_i}\times(\mathrm{PAF}_i)^{e_i}\right]\times\left[(1-\mathrm{PAF}_3)^{1-e_3}\times(\mathrm{PAF}_3)^{e_3}\right] \\ &= \prod_{i=1}^{3}(1-\mathrm{PAF}_i)^{1-e_i}\times(\mathrm{PAF}_i)^{e_i}.\end{aligned}$$

Repeating the process, we therefore have

$$\mathrm{CPW}_{e_1,\ldots,e_m} = \prod_{i=1}^{m}(1-\mathrm{PAF}_i)^{1-e_i}\times(\mathrm{PAF}_i)^{e_i},$$

for any positive integer m.
